# Clinical Outcomes in Children With Human Immunodeficiency Virus Treated for Nonsevere Tuberculosis in the SHINE Trial

**DOI:** 10.1093/cid/ciae193

**Published:** 2024-04-09

**Authors:** Chishala Chabala, Eric Wobudeya, Marieke M van der Zalm, Monica Kapasa, Priyanka Raichur, Robert Mboizi, Megan Palmer, Aarti Kinikar, Syed Hissar, Veronica Mulenga, Vidya Mave, Philippa Musoke, Anneke C Hesseling, Helen McIlleron, Diana Gibb, Angela Crook, Anna Turkova, the SHINE Trial Team, Louise Choo, Genevieve Wills, Margaret J Thomason, Jaqueline Teera, Ellen Owen-Powell, Kristen LeBeau, David Baptiste, Charlotte McGowan, Moira Spyer, Joyce Lungu, Kevin Zimba, Khozya Zyambo, Chalilwe Chungu, Chimuka Tembo, Sharon Kunda, Ellen Shingalili, Semy Zulu, Terence Chipoya, Habulembe Mwanakalanga, Elias Chambela, Jessy M Hankombo, Mox Malama Kalumbi, Daniel Chola, Stephen Malama, Winnie Nansamba, Mark Ssenyonga, Willy Ssengooba, Gerald Businge, Jessica Workman, Anne-Marie Demers, Simon Schaaf, Robert Gie, Elisabetta Walters, Warren Zimri, Graeme Hoddinott, Anneen van Deventer, Pierre Goussard, Julie Morrison, Aparna Nijampurkar, Sameer Khan, Bency Joseph, Perumal Kannabiran Bhavani, G Prathiksha, Dhanaraj Baskaran, N S Gomathi, V Mythily, Hemanth Kumar, Silambu Chelvi, L Sekar, Luke Hanna, K Ramesh, Hema Latha, S Bharathi, Parveen Banu, Dino Xavier, Manjith Kumar, K Guru, Sasi Kumar, A Kesavan, A Gunasundari, G Mangalambal, Valarmathi Nagarajan, Shakeela Shankar, R Selvi, S Vaishnavi, Krishna Yadav, R Supriya, Hema Giranab, A Seetha, Stella Mary, S Gopika, S Rohini, M Revathy, Sarath Balaji, S Elilarasi, J Ganesh, M A Aravind, Sylvia Mulambo, Hope Mwanyungwi, Dharati Tapse, Manasi Sane, Amina Abdullah, Sarah Nakalanzi, Cynthia Mukisa Williams, Rob Aarnoutse, Paul Revill, James Love-Koh, Simon Walker, Peter Mugyenyi, Janet Darbyshire, Polly Clayden, Peter Donald, Varinder Singh, Malgosia Grzemska, Soumya Swaminathan, Tim Peto, Alwyn Mwinga, Katherine Fielding, Stephen M Graham, Steven B Welch, James A Seddon, Elizabeth Whittaker, Suzanne Anderson, Louis Grandjean

**Affiliations:** Department of Paediatrics, School of Medicine, University of Zambia, Lusaka, Zambia; Children's Hospital, University Teaching Hospitals, Lusaka, Zambia; Faculty of Health Sciences, Department of Medicine, Division of Clinical Pharmacology, University of Cape Town, Cape Town, South Africa; Mulago Hospital, Makerere University–John Hopkins Hospital Research Collaboration, Kampala, Uganda; Department of Paediatrics and Child Health, Desmond Tutu TB Centre, Stellenbosch University, Cape Town, South Africa; Children's Hospital, University Teaching Hospitals, Lusaka, Zambia; Byramjee Jeejeebhoy Medical College, Johns Hopkins University Clinical Research Site, Pune, India; Mulago Hospital, Makerere University–John Hopkins Hospital Research Collaboration, Kampala, Uganda; Department of Paediatrics and Child Health, Desmond Tutu TB Centre, Stellenbosch University, Cape Town, South Africa; Byramjee Jeejeebhoy Medical College, Johns Hopkins University Clinical Research Site, Pune, India; Indian Council of Medical Research, National Institute for Research in Tuberculosis, Chennai, India; Department of Paediatrics, School of Medicine, University of Zambia, Lusaka, Zambia; Children's Hospital, University Teaching Hospitals, Lusaka, Zambia; Byramjee Jeejeebhoy Medical College, Johns Hopkins University Clinical Research Site, Pune, India; Mulago Hospital, Makerere University–John Hopkins Hospital Research Collaboration, Kampala, Uganda; Department of Paediatrics and Child Health, Desmond Tutu TB Centre, Stellenbosch University, Cape Town, South Africa; Faculty of Health Sciences, Department of Medicine, Division of Clinical Pharmacology, University of Cape Town, Cape Town, South Africa; Institute of Clinical Trials and Methodology, Medical Research Council–Clinical Trials Unit at University College London, London, United Kingdom; Institute of Clinical Trials and Methodology, Medical Research Council–Clinical Trials Unit at University College London, London, United Kingdom; Institute of Clinical Trials and Methodology, Medical Research Council–Clinical Trials Unit at University College London, London, United Kingdom

**Keywords:** tuberculosis, children with HIV, viral suppression, mortality, adverse events

## Abstract

**Background:**

Children with human immunodeficiency virus (HIV, CWH) are at high risk of tuberculosis (TB) and face poor outcomes, despite antiretroviral therapy (ART). We evaluated outcomes in CWH and children not living with HIV treated for nonsevere TB in the SHINE trial.

**Methods:**

SHINE was a randomized trial that enrolled children aged <16 years with smear-negative, nonsevere TB who were randomized to receive 4 versus 6 months of TB treatment and followed for 72 weeks. We assessed TB relapse/recurrence, mortality, hospitalizations, grade ≥3 adverse events by HIV status, and HIV virological suppression in CWH.

**Results:**

Of 1204 children enrolled, 127 (11%) were CWH, of similar age (median, 3.6 years; interquartile range, 1.2, 10.3 versus 3.5 years; 1.5, 6.9; *P* = .07) but more underweight (weight-for-age *z* score, −2.3; (3.3, −0.8 versus −1.0; −1.8, −0.2; *P* < .01) and anemic (hemoglobin, 9.5 g/dL; 8.7, 10.9 versus 11.5 g/dL; 10.4, 12.3; *P* < .01) compared with children without HIV. A total of 68 (54%) CWH were ART-naive; baseline median CD4 count was 719 cells/mm^3^ (241–1134), and CD4% was 16% (10–26). CWH were more likely to be hospitalized (adjusted odds ratio, 2.4; 1.3–4.6) and to die (adjusted hazard ratio [aHR], 2.6; 95% confidence interval [CI], 1.2 to 5.8). HIV status, age <3 years (aHR, 6.3; 1.5, 27.3), malnutrition (aHR, 6.2; 2.4, 15.9), and hemoglobin <7 g/dL (aHR, 3.8; 1.3,11.5) independently predicted mortality. Among children with available viral load (VL), 45% and 61% CWH had VL <1000 copies/mL at weeks 24 and 48, respectively. There was no difference in the effect of randomized treatment duration (4 versus 6 months) on TB treatment outcomes by HIV status (*P* for interaction = 0.42).

**Conclusions:**

We found no evidence of a difference in TB outcomes between 4 and 6 months of treatment for CWH treated for nonsevere TB. Irrespective of TB treatment duration, CWH had higher rates of mortality and hospitalization than their counterparts without HIV.

**Clinical Trials Registration.** ISRCTN63579542.

## Teaser

We compared outcomes between children with and without human immunodeficiency virus (HIV) treated for nonsevere TB. Regardless of treatment duration (4 or 6 months), children with HIV had similar TB outcomes but had higher mortality and hospitalization rates compared with their counterparts not living with HIV.

## Graphical abstract

**Graphical abstract:**
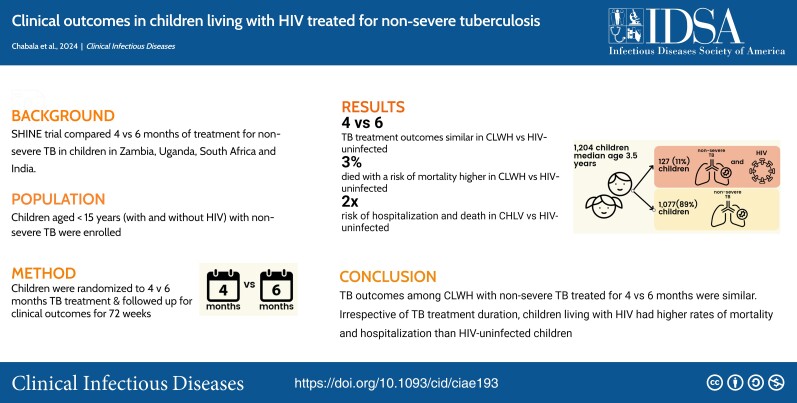
This graphical abstract is also available at Tidbit: https://tidbitapp.io/tidbits/clinical-outcomes-in-children-living-with-hiv-treated-for-non-severe-tuberculosis

Children with human immunodeficiency virus (HIV, CWH) are disproportionately affected by tuberculosis (TB). Annually, approximately 1.3 million children develop TB, resulting in 214 000 deaths; most cases go undiagnosed and untreated [1, 2]. Among children and adolescents aged <15 years who are dying from TB, 10% have been diagnosed with HIV [3]. Antiretroviral therapy (ART) confers significant benefits, reducing the incidence of TB in CWH [4, 5]. However, despite good immune recovery and viral suppression following ART initiation, the risk of developing TB remains higher in CWH compared with their counterparts without HIV [6], and ART may take up to 2 years to fully reach its potential for protection against TB [4].

Without ART, CWH have 5 times higher risk of death compared with their peers without HIV [5, 7, 8]. Diagnostic challenges hamper early identification of TB in CWH, leading to frequent late presentation with severe TB and advanced HIV disease, particularly in high TB endemic settings [9, 10]. HIV/TB coinfection is associated with worse HIV treatment outcomes and is among several factors that contribute to virological failure in individuals with HIV in sub-Saharan Africa [11]. Children concurrently treated for TB and HIV had 10% to 20% lower rates of viral suppression, with this risk persisting during the first year of ART treatment [12–14].

Treatment of TB in CWH is complicated by drug–drug interactions and overlapping toxicities between antiretroviral and rifampicin-based anti-TB regimens. In low- and middle-income countries, treatment options for children with HIV/TB are limited, and rifabutin, which is frequently used in high-income countries to substitute rifampicin, is not available. Overlapping toxicities from cotreatment increase the risk of adverse events (AEs) that may affect adherence and TB and HIV treatment outcomes [15–17].

In the SHINE trial, children with nonsevere TB, randomized to receive shorter (4-month) or standard (6-month) treatment, were followed up for 72 weeks and assessed for unfavorable outcomes [18]. The trial found that 4 months was noninferior to the standard 6 months of treatment in children with nonsevere TB [19]. Here, we report on clinical outcomes in CWH compared with children without HIV and describe HIV virological suppression at 24 and 48 weeks in CWH.

## METHODS

### Study Design and Population

The SHINE trial, a noninferiority trial, enrolled children aged <16 years with nonsevere TB following written informed consent from parents/guardians. Children of known HIV status with symptomatic, nonsevere, smear-negative, intrathoracic TB or peripheral lymphadenitis were included. Based on the Wiseman criteria [19, 20], nonsevere respiratory TB was defined as respiratory disease confined to a single lobe (opacification of <1 lobe) with none of the following on chest X ray (CXR): cavities, miliary disease pattern, complex pleural effusions, and significant airway compression. TB diagnosis was made by site clinicians based on clinical features, TB contact history, CXR findings, and mycobacterial test results.

We conducted a secondary analysis of TB treatment outcomes, mortality, hospitalizations, and grade ≥3AEs in CWH compared with children without HIV. TB treatment outcome was defined as unfavorable if any of the following occurred: death (all-cause), treatment failure, TB treatment change, restart or extension, TB recurrence, or loss to follow-up by 72 weeks [19]. Other clinical outcomes were death, hospitalization by 72 weeks, grade ≥3 AEs during and up to 30 days after treatment, and viral suppression at 24 and 48 weeks in CWH. AE severity was graded following established criteria [21].

### Study Procedures and Interventions

Eligible children with presumptive TB were screened and had a clinical evaluation including TB symptoms, contact history, and a physical examination. Details of TB evaluation and initiation procedures are published elsewhere [18, 19]. At enrollment, respiratory samples and/or lymph node aspirates (if clinically indicated) were obtained for smear microscopy, Xpert MTB/RIF (Xpert, Cepheid), and mycobacterial culture. CXRs were performed to inform diagnostic certainty and TB disease severity classification and to establish trial eligibility. In addition, using standard criteria [18, 22], a separate independent endpoint review committee (ERC), blinded to treatment allocation, reviewed clinical events suggestive of treatment failure or TB recurrence and all deaths.

At enrollment, blood samples were obtained for hematology and chemistry in all children, and CD4 cell count and HIV-1 viral load (VL) testing (if it was part of the standard of care in the country) were obtained for CWH. Children were seen at week 2, every 4 weeks until week 28, and then every 3 months up to week 72. At each visit, clinical evaluations were performed to detect new AEs, treatment failures, or recurrence of TB. CD4 cell count was repeated at weeks 24 and 48. HIV-1 VL was repeated at the intervals specified by national recommendations. Acute illnesses, hospitalizations, and deaths were documented throughout follow-up.

Anti-TB treatment (rifampicin, isoniazid, pyrazinamide, and/or ethambutol) was administered according to randomization arm (4 or 6 months duration) and dosed per World Health Organization (WHO)–recommended weight bands using fixed-dose combination tablets (Macleods Pharmaceuticals, India) [23].

ART was provided in accordance with WHO 2016 treatment guidelines [24] and respective national recommendations. ART included 2 nucleoside reverse-transcriptase inhibitors (lamivudine with abacavir, zidovudine, or tenofovir disoproxil fumarate) plus efavirenz (EFV) or lopinavir/ritonavir (LPV/r). For anti-TB cotreatment, LPV/r was superboosted (if single-formulated ritonavir was available) or the daily dose of LPV/r was doubled.

### Statistical Analyses

Baseline characteristics were compared using *χ*^2^ and *t* tests. Heterogeneity of the effect of the randomized duration of treatment on TB outcomes assessed at the end of treatment was formally tested between CWH and children without HIV by inclusion of a treatment by HIV status interaction term in a binary risk difference model. The interaction was tested by use of a likelihood ratio test to compare models with and without the interaction term, with *P* < .1 considered evidence of a different effect (4 versus 6 months) between CWH and children without HIV. For this comparison, we included outcomes from week 16 onward (before week 16, treatment in the randomized groups was the same).

Logistic regression and Cox proportional hazards (CPH) models were fitted to investigate baseline predictors of clinical outcomes including age, sex, randomization arm, clinical sites, weight-for-age *z* score (WAZ), and TB disease characteristics in CWH versus children without HIV. Mortality was fitted as a time-to-event outcome (CPH models, summary measure hazard ratio [HR]); other outcomes were fitted as binary (logistic regression, summary measure odds ratio [OR]). All multivariable models included HIV status, age, country, TB confirmation, and randomized duration of treatment. Other factors found to be significant (*P* < .1) in the univariable model were also included.

In the CWH cohort, timing of ART, ART regimens, and viral suppression <1000 mL copies/mL at 24 and 48 weeks were assessed.

### Ethical Considerations

The study was approved by the ethics committees and regulatory authorities in Zambia, Uganda, South Africa, and India and by the sponsor, the University College London Ethics Committee.

## RESULTS

Of the 1204 children enrolled in the trial, 127 (11%) were CWH, the majority from Zambia (91, 72%) and Uganda (31, 24%; Table 1). Of CWH, two-thirds (65%) had advanced or severe HIV (before the TB episode); median (interquartile range [IQR]) baseline CD4% and CD4 cell counts were 16% (10, 26) and 719 cells/mm^3^ (241, 1134), respectively. At enrollment, 68 (54%) of the CWH were ART-naive (Table 2). Baseline HIV1 VL was available for 23 children; median (IQR) baseline HIV1 VL was 89 973 (1864, 587 977); and 4 (17%) had VL <1000. Most started ART within 2–8 weeks after starting TB treatment; 2 died before ART initiation, and ART status data were missing for 9.

**Table 1.** Baseline Characteristics of Participants by Human Immunodeficiency Virus Status

Characteristic	Diagnosed with HIV (n = 127)	Not Diagnosed with HIV (n = 1077)	Total (n = 1204)	*P* Value^a^
Age, y	3.6 (1.2, 10.3)	3.5 (1.5, 6.9)	3.5 (1.5,7.0)	.07
Female	63 (49.6)	520 (48.3)	583 (48.4)	.78
Weight-for-age zscore	−2.3 (−3.3, −0.8)	−1.0 (−1.8, −0.2)	−1.0 (−1.9, 0.3)	<.01 (t test)
Hemoglobin count, g/dL	9.5 (8.7, 10.9)	11.5 (10.4, 12.3)	11.3 (10.1, 12.2)	<.01 (t test)
Alanine aminotransferase	20.2 (13.8, 35.2)	15.0 (12.0, 20.8)	15.7 (12.0, 21.4)	<.01 (t test)
Site of disease,^b^ n (%)				
Pulmonary only	74 (58.3)	730 (67.8)	804 (66.8)	<.01
Pulmonary and lymph node	53 (41.7)	300 (27.9)	353 (29.3)	…
Lymph node only	0 (0)	40 (3.7)	40 (3.3)	…
Other	0 (0)	7 (0.7)	7 (0.6)	…
TB status				
Bacteriologically confirmed	8 (6.3)	157 (14.6)	165 (13.7)	.01
Unconfirmed	95 (74.8)	698 (64.1)	1039 (65.9)	…
Unlikely	24 (18.9)	222 (20.6)	246 (20.4)	…
Anti-TB drug dosing weight band, kg				
3.0 ≤ 3.9	2 (1.6)	1 (0.1)	3 (0.3)	<.01
4.0 ≤ 7.9	35 (27.6)	145 (13.5)	180 (15.0)	…
8.0 ≤ 11.9	29 (22.8)	284 (26.4)	313 (26.0)	…
12.0 ≤ 15.9	10 (7.9)	231 (21.5)	241 (20.0)	…
16.0 ≤ 24.9	31 (24.4)	266 (24.7)	297 (24.7)	…
≥25.0	20 (15.8)	150 (13.9)	170 (14.1)	…
Site/Country				
Zambia	91 (71.6)	273 (25.3)	364 (30.2)	<.001
Uganda	31 (24.4)	345 (32.0)	376 (31.2)	…
South Africa	5 (3.9)	310 (28.8)	315 (26.2)	…
India	0 (0)	149 (13.8)	149 (12.4)	…
Randomization arm				
4 m	65 (51.2)	537 (49.9)	602 (50.0)	…
6 m	62 (48.8)	540 (50.1)	602 (50.0)	…

Data presented as number of participants (%) or median (interquartile range).

Abbreviations: HIV, human immunodeficiency virus; TB, tuberculosis.

^a^ *P* value from *χ*^2^ test unless otherwise stated.

^b^Intrathoracic lymph node was classified as pulmonary TB; peripheral lymph node disease without any respiratory symptoms or abnormal chest X-ray findings was classified as lymph node disease.

**Table 2.** Clinical Characteristics of Participants With Human Immunodeficiency Virus

Clinical Characteristic (n = 127)	Frequency, n (%)
Human immunodeficiency virus treatment status at baseline	
Naive	68 (53.5)
On ART	59 (46.5)
World Health Organization immunological classification^a^ (n = 88)	
Not significant	21 (24.4)
Mild	8 (9.3)
Advanced	8 (9.3)
Severe	49 (57.0)
CD4 count (n = 95), cell/mm^3^	
<200	19 (20.0)
≥200–350	10 (10.5)
≥350–500	7 (7.4)
≥500	59 (62.1)
CD4% (n = 86)	
<15	34 (39.5)
≥15	52 (60.5)
ART regimen (n = 116)	
Efavirenz-based	60 (51.7)
Lopinavir-based	43 (37.1)
Nevirapine-based	13 (11.2)

Abbreviation: ART, antiretroviral therapy.

^a^World Health Organization case definitions of human immunodeficiency virus (HIV) for surveillance and revised clinical staging and immunological classification of HIV-related disease in adults and children [25].

CWH were of similar median (IQR) age compared with children without HIV (3.6 years, 1.2, 10.3 versus 3.5 years, 1.5–6.9 years) but were more underweight (WAZ: −2.3; −3.3, −0.8 versus −1.0; −1.8, −0.2; *P* < .001) and had lower hemoglobin counts (9.5 g/dL, 8.7, 10.9 versus 11.5 g/dL, 10.4, 12.3; *P* < .001.). CWH had a higher proportion of mixed (pulmonary and lymph node) TB disease (42% versus 28%, *P* < .001) compared with children without HIV. Overall, 165 (14%) children had bacteriologically confirmed TB by Xpert *Mycobacterium tuberculosis*/rifampin or culture, with a significantly smaller proportion (8, 6%) in CWH compared with children without HIV (165, 15%; *P* <.01; Table 1).

Overall, 92% of the children in the trial had favorable TB treatment outcomes. In the modified intention-to-treat population, the adjusted absolute risk difference of an unfavorable outcome (4-month versus 6-month arm) was −4.3% (in favor of 4-months, 95% confidence interval [CI], −14.9 to 6.2 in CWH) and 0.0% (95% CI, −1.8 to 1.8) in children without HIV, with no heterogeneity in the effect of randomized duration of treatment (4 versus 6 months of antituberculosis treatment) on TB treatment outcomes by HIV status (test for interaction, *P* = .42; Table 3).

**Table 3.** Subgroup Analysis of Tuberculosis Outcomes and Randomized Treatment Duration by Human Immunodeficiency Virus Status

	Children With HIV		Children Not Diagnosed With HIV		
Subgroup parameters assessed	4 Months	6 Months	4 Months	6 Months	Total
Total randomized	65	62	537	540	1204
Unassessable^a^	6	8	24	21	59
Did not reach week 16	4	5	11	11	31
Favorable	55 (93%)	48 (89%)	501 (98%)	507 (98%)	1111 (97%)
Unfavorable	4 (7%)	6 (11%)	12 (2%)	12 (2%)	34 (3%)
Total assessable	59	54	513	519	1145
* *Difference from control in unfavorable rate	− 4.3%	…	0.03%	…	…
* *95% confidence interval	(−14.9 to 6.2)	…	(−1.8 to 1.9)	…	…

Test for interaction: *P* value = .42. Test for interaction compares the effects (risk difference) of treatment duration on tuberculosis (TB) outcomes, comparing children with HIV and children not diagnosed with HIV.

Abbreviation: HIV, human immunodeficiency virus.

^a^Reasons for unassessable include not reaching week 16 and late exclusions for drug-resistant TB.

Unfavorable TB treatment events were more frequent in CWH (n = 24, 19%) compared with children without HIV (n = 69, 6%; adjusted HR [aHR], 2.4; 1.3, 4.2; Table 4). Across both groups, in addition to HIV status, age <3 years (aHR, 3.0; 95% CI, 1.7 to 5.2), being underweight (WAZ, ≤−2; aHR, 1.8; 95% CI, 1.1 to 2.9), and anemic hemoglobin <7g/dL; aHR, 4.6; 95% CI, 2.1 to 10.3) were independently predictive of unfavorable TB outcomes (Supplementary Table 1).

**Table 4.** Mortality, Hospitalizations, and Nonfatal Grade ≥3 Adverse Events by Human Immunodeficiency Virus Status

Clinical Outcome	Living With HIV n = 127	Not Living With HIV n = 1077	Adjusted Hazard Ratio^a^ (95% CI)
Tuberculosis treatment outcome			
Unfavorable	24 (18.9)	69 (6.4)	2.4 (1.3 to 4.2)
Favorable	103 (81.1)	1008 (93.6)	…
Mortality			
Dead	13 (10.2)	18 (1.7)	2.6 (1.2 to 5.8)
Alive	114 (89.8)	1059 (98.3)	
			Adjusted Odds Ratio^b^ (95% CI)
Hospitalization			
Yes	18 (14.2)	95 (8.8)	2.4 (1.3 to 4.6)
No	109 (85.8)	982 (91.2)	…
Adverse event (grade ≥3)			
Yes	24 (18.9)	71 (6.6)	4.4 (2.3 to 8.5)
No	103 (81.1)	982 (93.4)	…

Abbreviations: CI, confidence interval; HIV, human immunodeficiency virus.

^a^Adjusted for age, hemoglobin count, weight-for-age z score, bacteriological confirmation, country, and randomized tuberculosis (TB) treatment duration.

^b^Adjusted for age, weight-for-age z score, bacteriological confirmation, site of TB disease, hemoglobin count, country, and randomized TB treatment duration.

Of 113 children with at least 1 hospitalization in 72 weeks of follow-up, 18 (14%) were among CWH compared with 95 (9%) among children without HIV. Overall, the risk of hospitalization was more than twice as high among CWH compared with children without HIV (aHR, 2.4; 95% CI, 1.3 to 4.6; Table 4). In both groups, age <3 years (aOR, 1.8; 95% CI, 1.2 to 2.9) also predicted hospitalization (Supplementary Table 1).

Thirty-one of the 1204 (3%) deaths in the trial (Uganda, 17 of 376 (5%); Zambia, 13 of 364 (4%); South Africa, 1 (<1%) of 315; India, 0 (0%) of 149; 12 (39%) occurred before week 16. Thirteen of 127 (10%) were among CWH and 18 of 1077 (2%) were among children without HIV (aHR, 2.6; 95% CI, 1.2 to 5.8). Pneumonia (n = 6) followed by seizures (n = 2), septicemia (n = 2), diarrheal disease (n = 2), and hypotension/shock and trauma (n = 3) were the common causes of death (Supplementary Table 2). As adjudicated by the ERC, blinded to trial arm, 7 of 13 (54%) deaths among CWH and 6 of 18 (33%) among children without HIV were due to TB. The median (IQR) time to death was 4.6 months (2.1, 7.0) shorter in CWH than in children without HIV (6.6 months, 1.8, 8.8; Table 4, Supplementary Figure 1). Independent of HIV status, age <3 years (aHR, 6.3; 95% CI, 1.5 to 27.3), being underweight (aHR, 6.2; 95% CI, 2.4 to 15.9), and being anemic (aHR, 3.8; 95% CI, 1.3 to 11.5) had higher risks of mortality (Supplementary Table 1).

Seventy-four children experienced at least 1 grade 3 or 4 AE, reported up to 30 days after the last dose of TB treatment. Grade ≥3 AEs reported in ≥5% were pneumonia or chest infection (29, 25%), deranged liver function (11, 10%), diarrheal diseases (7, 6%), and epilepsy or seizures (6, 5%). CWH were more likely to experience a grade ≥3 AE compared with children without HIV (aHR, 4.6; 95% CI, 2.1 to 9.9; Table 4). In both groups, children aged <3 years had a higher risk of experiencing a grade ≥3 AE than older children (aOR, 2.1; 95% CI, 1.2 to 3.6; Supplementary Table 1).

HIV-1 VL results were available for 65 of 119 (55%) and 82 of 115 (71%) CWH at week 24 and at week 48, respectively; 45% (week 24) and 61% (week 48) had VL <1000 copies/mL. A higher proportion of children who received EFV-based ART were virologically suppressed compared with those on LPV/r-based ART at week 48 (29 of 41, 71%) versus 20 of 40 (50%; *P* = .056).

## DISCUSSION

Our findings show that overall unfavorable TB treatment outcomes were more frequent in CWH compared with children without HIV. However, when randomized duration of TB treatment was compared, there was no difference in unfavorable outcomes between 4 and 6 months in CWH. Thus, there was no evidence to suggest that CWH with nonsevere TB needed longer treatment duration than children without HIV and that both groups can receive the shorter 4 months of treatment. CWH were at higher risk of hospitalizations and grade ≥3 AEs compared with those without HIV. Mortality was infrequent in the entire study population; however, as expected, the risk was higher in CWH, and time to death was sooner. Viral suppression in CWH was suboptimal with only 61% having VL <1000 at 48 weeks. Children on LPV/r-based ART had a trend toward worse VL responses compared with those on EFV-based regimens.

Our results concur with those from the many pediatric studies that have reported worse TB treatment outcomes in CWH compared with children without HIV, particularly in the presence of severe TB disease [26–31]. We enrolled only children with nonsevere TB, and despite overall excellent treatment outcomes, HIV coinfection increased the risk of unfavorable TB outcomes, as observed in cohorts with a wider spectrum of disease [7, 8, 26, 27]. Similarly, the risks of hospitalization and death were significantly higher in CWH. Despite this, the rate of mortality in the SHINE study was similar to or lower than the reported mortality in children below 5 years in the respective countries in 2020 (India, 4%; South Africa, 3%; Uganda, 4%; and Zambia, 6%) [32]. Among CWH, >10% died compared with <2% in children without HIV. Mortality rates for children with TB vary widely, from <1% in the Netherlands to 13% in Nigeria, with higher rates reported in children with severe forms of TB [7, 8, 26–29, 33]. Observational studies of children with HIV/TB coinfection have reported higher mortality rates of 10% to 20%, with worse outcomes in ART-naive populations [5, 9, 10, 34]. In our cohort, less than half of the CWH were on ART at the time of TB diagnosis and more than 80% were on ART by the end of the intensive phase. While higher ART coverage reduces the risk of mortality in CWH with TB, it does not eliminate it [35, 36]. Deaths in individuals with TB often occur in the first few months of treatment, particularly in those with HIV where rapid progression of TB can occur even in the absence of immune compromise [35–37].

There were few grade ≥3 AEs observed in the trial. However, CWH had a 5-fold risk of experiencing an AE. After chest infections, elevation of liver enzymes was the most common treatment-related AE [19]. Transient and asymptomatic transaminitis is commonly observed in children on TB treatment and represents hepatic adaptation to TB treatment and is often reversible [38].

Our study showed that children aged <3 years, with or without HIV, faced a significant risk of experiencing unfavorable TB outcomes, hospitalization, death, and AEs. Of note, among CWH, a higher proportion were underweight and anemic compared with those without HIV. Being underweight was an independent predictor of poor clinical outcomes, emphasizing the critical role of inadequate nutrition in contributing to unfavorable outcomes regardless of HIV status in children with TB. Younger age and undernutrition were previously identified as risk factors for TB-associated mortality. Our study emphasizes that this remains relevant even in children with nonsevere TB disease [35, 39, 40]. Furthermore, anemia (hemoglobin <7g/dL) independently predicted unfavorable TB outcomes, hospitalizations, and mortality, irrespective of HIV status. Anemia is frequently observed in children from low- and middle-income countries, often associated with undernutrition [41], and it commonly complicates HIV [42]. Notably, it is a prevalent comorbidity with TB and linked to poor TB treatment outcomes [43, 44].

Among CWH with available HIV-1 VLs, viral suppression was suboptimal after 1 year of follow-up. This could be due to insufficient duration of ART since only half of the children were established on ART at the time of enrollment and ART initiation times varied. Previous studies suggested that children cotreated for TB around the time of ART initiation had low rates of virological suppression [12–14, 45]. Another possible reason could be biased selective targeting of VL testing in children with suspected nonadherence to ART. We observed a trend toward better viral suppression in children who received EFV-based ART compared with those on LPV/r-based ART. South African studies reported lower rates of viral suppression in children who received ritonavir-boosted LPV/r-based ART who were cotreated for TB [13, 14]. For children aged <3 years, superboosted LPV/r is the preferred strategy to overcome the interaction with rifampicin. However, not all sites implemented LPV/r superboosting during the SHINE trial due to the nonavailability of single-formulated ritonavir. In Zambia, double-dosing of LPV/r or switching to EFV in children aged ≥3 years was practiced for rifampicin cotreatment in the absence of single-formulated ritonavir. However, LPV/r double-dosing was previously associated with suboptimal lopinavir concentrations with rifampicin cotreatment [46]. Drug resistance mutations to protease inhibitors are infrequent despite virological failures, suggesting that other treatment-related factors (in particular, adherence difficulties) are likely the main contributors to nonviral suppression [47], especially since pediatric formulations of LPV/r are not palatable and require twice-daily administration [13, 48, 49]. The currently recommended dolutegravir (DTG)-based ART, which is associated with high rates of viral suppression and excellent safety, is considered the preferred treatment option for CWH with HIV/TB despite requiring twice-daily dosing in children with HIV/TB [50]. DTG was not available for pediatric use at the time of the trial.

This study has several limitations including lack of generalizability to children with severe TB disease. The trial aimed to evaluate the noninferiority of 4 months versus 6 months of treatment in children with nonsevere TB; therefore, CWH with severe TB were excluded. Second, assessment of viral suppression in CWH may have been affected by missing VL results. In this pragmatic trial, VLs were measured per national recommendations, which did not mandate frequent VL testing. Nevertheless, our findings align with observations in other cohorts that suggest suboptimal VL suppression in CWH diagnosed with TB and starting ART [12, 13]. Despite these limitations, this trial had a considerable number of CWH and excellent follow-up of up to 72 weeks, allowing comprehensive documentation of the clinical outcomes. TB diagnoses were thoroughly evaluated using well-defined criteria and independent expert review [20, 22].

We found no evidence of a difference between 4 and 6 months of treatment for CWH being treated for nonsevere TB. Acknowledging that the interaction test was underpowered and the analysis can only be considered exploratory, it was nevertheless reassuring that along with all the other subgroup analyses performed, the results were consistent with the overall trial findings [19]. Benefits of shortening TB treatment include improved treatment adherence, minimal losses to follow-up, and fewer AEs associated with prolonged TB treatment. Efforts should focus on equipping health services and providers with the capacity to appropriately determine TB disease severity and identify children who can benefit from the shorter regimen. In addition, patient-centered management should include the identification of potential risk factors, such as anemia and undernutrition, that are likely to impact treatment outcomes. Early HIV diagnosis, access to ART, and comprehensive patient care that includes nutritional support can improve outcomes and reduce the risk of poor outcomes in CWH.

## CONCLUSIONS

In conclusion, our study highlights that CWH with nonsevere TB had a higher risk of poor clinical outcomes compared with children without HIV. We found no significant differences in TB treatment outcomes between CWH and children without HIV who received 4 versus 6 months of TB treatment, providing reassurance that CWH can receive shorter 4 months of TB treatment if they have nonsevere TB.

## Supplementary Data

Supplementary materials are available at *Clinical Infectious Diseases* online. Consisting of data provided by the authors to benefit the reader, the posted materials are not copyedited and are the sole responsibility of the authors, so questions or comments should be addressed to the corresponding author.

## Supplementary Material

ciae193_Supplementary_Data

## Notes

***Acknowledgments.*** The authors thank the children who participated in the SHINE trial and their caregivers; the nursing, pharmacy, and laboratory staff; all those who advised, volunteered, or otherwise supported community engagement at the trial sites; and the members of the trial steering committee, the endpoint review committee, and the independent data and safety monitoring committee for their contributions, including oversight of the safety of the trial.

SHINE Trial Team:

*SHINE Trial Team UK Medical Research Council, Clinical Trials Unit at University College London:* Louise Choo, Genevieve Wills, Margaret J. Thomason, Jaqueline Teera, Ellen Owen-Powell, Kristen LeBeau, David Baptiste, Charlotte McGowan, Moira Spyer. *University Teaching Hospital, Children's Hospital, Lusaka, Zambia:* Joyce Lungu, Kevin Zimba, Khozya Zyambo, Chalilwe Chungu, Chimuka Tembo, Sharon Kunda, Ellen Shingalili, Semy Zulu, Terence Chipoya, Habulembe Mwanakalanga, Elias Chambela, Jessy M. Hankombo, Mox Malama Kalumbi, Daniel Chola, Stephen Malama. *Makerere University–Johns Hopkins University Research Collaboration, Kampala, Uganda:* Winnie Nansamba, Mark Ssenyonga, Willy Ssengooba, Gerald Businge. *Desmond Tutu TB Centre, Stellenbosch University, South Africa:* Jessica Workman, Anne-Marie Demers, Simon Schaaf, Robert Gie, Elisabetta Walters, Warren Zimri, Graeme Hoddinott, Anneen van Deventer, Pierre Goussard, Julie Morrison. *Byramjee Jeejeebhoy Government Medical College, Pune, India:* Aparna Nijampurkar, Sameer Khan. *Indian Council of Medical Research, National Institute for Research in Tuberculosis, Chennai, India:* Bency Joseph, Perumal Kannabiran Bhavani, G. Prathiksha, Dhanaraj Baskaran, N. S. Gomathi, V. Mythily, Hemanth Kumar, Silambu Chelvi, L. Sekar, Luke Hanna, K. Ramesh, Hema Latha, S. Bharathi, Parveen Banu, Dino Xavier, Manjith Kumar, K. Guru, Sasi Kumar, A. Kesavan, A. Gunasundari, G. Mangalambal, Valarmathi Nagarajan, Shakeela Shankar, R. Selvi, S. Vaishnavi, Krishna Yadav, R. Supriya, Hema Giranab, A. Seetha, Stella Mary, S. Gopika, S. Rohini, M. Revathy. *Institute of Child Health and Hospital for Children, Chennai, India:* Sarath Balaji, S. Elilarasi. *Government Stanley Medical College and Hospital, Chennai, India:* J. Ganesh, M. A. Aravind. *Local site monitors:* Sylvia Mulambo, Hope Mwanyungwi, Dharati Tapse, Manasi Sane, Amina Abdullah, Sarah Nakalanzi, Cynthia Mukisa Williams. *Radboud University Medical Center, Nijmegen, the Netherlands:* Rob Aarnoutse. *University of York, York, United Kingdom:* Paul Revill, James Love-Koh, Simon Walker. *SHINE Trial Steering Committee members:* Peter Mugyenyi (chair), Janet Darbyshire, Polly Clayden, Peter Donald, Varinder Singh, Malgosia Grzemska (replaced by Sabine Verkuijl and Annemieke Brands in March 2020), Soumya Swaminathan. *SHINE Independent Data Monitoring Committee members:* Tim Peto (Chair), Alwyn Mwinga, Katherine Fielding. *SHINE Endpoint Review Committee members:* Stephen M. Graham (Chair), Steven B. Welch, James A. Seddon, Elizabeth Whittaker, Suzanne Anderson, Louis Grandjean.

***Financial support*.** This work was supported by a grant (MR/L004445/1) from the Medical Research Council (UK MRC), the Department for International Development, the Wellcome Trust, National Institute for Health Research (UK) under the Joint Global Health Trials Scheme. Additional support was by the UK MRC (core support grant; MC_UU_12023/26 and MC_UU_12023/27). TB Alliance provided support for trial drugs purchases.
