# Supplementary material for: Clinical Outcomes in Children With Human Immunodeficiency Virus Treated for Nonsevere Tuberculosis in the SHINE Trial
Source: Clin Infect Dis. 2024 Apr 9;79(1):70–7. doi: 10.1093/cid/ciae193 (PMC11259218; doi:10.1093/cid/ciae193)
Supplement: ciae193_Supplementary_Data [file ciae193_supplementary_data.docx]

**Supplemental Figure and tables**

**Supplemental figure:**

Figure 1. Time to death by HIV status

Supplemental table 1. Mortality, TB treatment outcomes, hospitalizations, and occurrence of non-fatal grade ≥3 adverse events by patient characteristics

| **Patients’ characteristics** | **Mortality** | | **AHR***  **(95% CI)** | **TB treatment outcomes** | | **AHR***  **(95% CI)** | **Hospitalizations** | | **AOR****  **(95% CI)** | **Non-fatal Grade ≥3 AEs** | | **AOR****  **(95% CI)** |
| --- | --- | --- | --- | --- | --- | --- | --- | --- | --- | --- | --- | --- |
|  | Dead  n=31 | Alive  n=1173 |  | Unfavorable  n=93 | Favorable  n=1111 |  | Yes  n=113 | No  n=1091 |  | Yes  n=74 | No  n=1099 |  |
| **Age group (years)** |  |  |  |  |  |  |  |  |  |  |  |  |
| 3 and above | 27 (87.1) | 516 (44.0) | 6.4(1.5,27.3) | 64(68.8) | 479(43.1) | 3.0(1.7, 5.2) | 70(62.0) | 473(43.4) | 1.8(1.2,2.9) | 47(63.5) | 469(42.7) | 2.1(1.2,3.6) |
| Below 3 | 4 (12.9) | 657 (56.0) |  | 29(31.2) | 632(56.9) |  | 43(38.0) | 618(56.1) |  | 27(36.5) | 630(57.3) |  |
| **Sex** |  |  |  |  |  |  |  |  |  |  |  |  |
| Female | 15 (48.4) | 568 (48.4) | - | 52(55.9) | 531(47.8) | - | 52(46.0) | 531(48.7) |  | 39(48.4) | 529(48.1) | - |
| Male | 16 51.6) | 605 (51.6) |  | 41(44.1) | 580(52.2) |  | 61(54.0) | 560(51.3) |  | 35(47.3) | 570(51.9) |  |
| **Weight-for-age zscore** |  |  |  |  |  |  |  |  |  |  |  |  |
| >-2 | 23(79.3) | 226(22.4) | 6.2(2.4,15.9) | 37(45.1) | 212(22.2) | 1.8(1.1,2.9) | 35(32.7) | 214(23.0) | 1.1 (0.7, 1.9) | 21(30.0) | 205(21.9) | 1.1(0.6, 2.0) |
| ≤-2 | 6(20.7) | 782(77.6) |  | 45(54.9) | 743(77.8) |  | 72(67.3) | 716 (77.0) |  | 49(70.0) | 773(78.1) |  |
| **Site of disease (n, %)** |  |  |  |  |  |  |  |  |  |  |  |  |
| Pulmonary | 22(71.0) | 782(66.7) | - | 60(64.5) | 744(67.0) | - | 83(73.4) | 721(66.0) | 0.5(0.3,0.8) | 54(72.9) | 728(66.2) | 0.6(0.4, 1.2) |
| Mixed | 9(29.0) | 344(29.3) |  | 29(31.2) | 324(29.2) |  | 24(6.8) | 329(30.1) |  | 18(24.3) | 326(29.7) |  |
| Lymph node disease | 0(0) | 40(3.4) |  | 3(3.2) | 37(3.3) |  | 5(4.4) | 35(3.2) |  | 1(1.3) | 39(3.6) |  |
| Other | 0(0) | 7(0.6) |  | 1(1.1) | 6(0.5) |  | 1(0.9) | 6(0.6) |  | 1(1.3) | 6(0.6) |  |
| **Tuberculosis status** |  |  |  |  |  |  |  |  |  |  |  |  |
| Confirmed | 0 (0) | 165 (14.7) | - | 78(83.9) | 961(86.5) | 1.4(0.8, 2.9) | 101(89.3) | 938(86.0) | 0.9(0.4,1.7) | 10(13.5) | 155(14.1) | 1.2(0.6,2.0) |
| Unconfirmed | 31 (100) | 1,008 (85.9) |  | 15(16.1) | 150(13.5) |  | 12(10.6) | 153(14.0) |  | 64(86.5) | 944(85.9) |  |
| **Anti-TB dosing weight band** (Kg) |  |  |  |  |  |  |  |  |  |  |  |  |
| 3.0 to 7.9 | 24(74.2) | 159(13.6) | - | 39(41.9) | 144(13.0) | - | 30(26.6) | 153(14.0) | - | 19(25.6) | 140(12.7) | - |
| 8.0 ≤ 24.9 | 6(19.3) | 845(72.0) |  | 48(51.6) | 803(72.3) |  | 76(67.2) | 775(71.0) |  | 51(68.9) | 794(77.2) |  |
| ≥25.0 | 1(3.2) | 169(14.4) |  | 6(6.5) | 164(14.8) |  | 7(6.2) | 163(14.9) |  | 4(5.4) | 165(15.0) |  |
| **Haemoglobin (g/dL)** |  |  |  |  |  |  |  |  |  |  |  |  |
| ≥7 | 27 (87.0) | 1,160 (98.9) | 3.8(1.3,11,4) | 86(92.5) | 1101(99.1) | 4.6(2.1,10.3) | 108(95.6) | 1,079(98.9) | 2.7(0.8,9.0) | 72(98.9) | 1088(99.0) | 1.1(0.1,9.4) |
| <7 | 4 (12.9) | 13 (1.1) |  | 7(7.5) | 10(0.9) |  | 5(4.2) | 12(1.1) |  | 2(2.7) | 84.6(1.0) |  |
| **Site/country** |  |  |  |  |  |  |  |  |  |  |  |  |
| Zambia | 13 (41.9) | 351 (29.9) | *** | 37(39.8) | 327(29.4) | 1.6(0.7,3.7) | 34(30.1) | 330(30.3) | 0.5(0.2,1.0) | 22(29.7) | 329(29.9) | 0.9(0.4,2.1) |
| Uganda | 17 (54.8) | 359 (30.6) |  | 28(30.1) | 348(31.3) | - | 29(25.7) | 347(31.8) | 0.3(0.1,0.6)- | 19(25.7) | 340(30.9) | 0.5(0.2,1.1) |
| South Africa | 1 (3.2) | 314 (26.7) | - | 16(17.2) | 299(26.9) | 0.8(0.4,1.6) | 32(28.3) | 283(25.9) | 0.3(0.1,0.6) | 25(33.8) | 289(26.3) | 0.43(0.2.1.1) |
| India | 0 (0) | 149 (12.7) | - | 12(12.9) | 137(12.3) | 1.3(0.8,2.2) | 18(15.9) | 131(12.0) | - | 8(10.8) | 141(12.8) | - |
| **Randomized treatment duration** |  |  |  |  |  |  |  |  |  |  |  |  |
| 4 months | 12(38.7) | 590(50.3) | 1.3(0.6,2,7) | 46(49.5) | 556(50.1) | 0.9(0.6,1.5) | 60(53.1) | 542(49.7) | 0.8(0.5,1.2) | 38(51.3) | 552(50.2) | 0.9(0.6,1.5) |
| 6 months | 19(61.3) | 583(49.7) |  | 47(50.5) | 555(49.9) |  | 53(46.9) | 549(50.3) |  | 36(48.7) | 547(49.8) |  |
|  |  |  |  |  |  |  |  |  |  |  |  |  |

Values are n (%) unless otherwise specified.

*Adjusted for age, hemoglobin count, weight-for-age z-score, bacteriological confirmation, study site and randomized TB treatment duration.

**Adjusted for age, weight-for-age z-score, site of tuberculosis disease, hemoglobin count and study site.

***estimates not possible due to small number of events.

Supplemental table 2: Cause of death by the HIV status

| **Cause of death** | **HIV+** | **HIV-** | **Total** |
| --- | --- | --- | --- |
| Pneumonia | 4 | 2 | 6 |
| Epilepsy/ convulsions | 0 | 2 | 2 |
| Diarrhea (acute or chronic) | 2 | 0 | 2 |
| Severe malnutrition | 1 | 0 | 1 |
| Septicaemia | 1 | 1 | 2 |
| Congestive heart failure | 0 | 1 | 1 |
| Anemia | 1 | 0 | 1 |
| Acute respiratory failure | 1 | 0 | 1 |
| Hypotension/shock | 0 | 2 | 2 |
| Unknown | 3 | 5 | 8 |
| trauma | 0 | 3 | 3 |
| acute asthma | 0 | 1 | 1 |
| solid tumour | 0 | 1 | 1 |
| **Total** | **13** | **18** | **31** |

*An independent endpoint review committee, blinded to treatment allocation, reviewed all clinical and relevant data for all deaths and ascribed causes of death based on the data available.
